# Vitamin and dietary supplements are not associated with total or cardiovascular mortality in Switzerland: the CoLaus|PsyCoLaus prospective study

**DOI:** 10.1007/s00394-025-03593-1

**Published:** 2025-02-01

**Authors:** Rosa Lourenço, Pedro-Marques Vidal

**Affiliations:** 1 University of Lausanne, Lausanne, Switzerland; 2 Department of Medicine, Internal Medicine, Lausanne University Hospital (CHUV) and University of Lausanne, Office BH10-642, Rue du Bugnon 46, 1011 Lausanne, Switzerland

**Keywords:** Mortality, Cardiovascular disease, Vitamin supplements, Prospective study

## Abstract

**Purpose:**

Vitamin-mineral and dietary supplements (VMDS) are taken by a large fraction of the population. Whether their long-term consumption impacts mortality and cardiovascular disease (CVD) has seldom been studied.

**Methods:**

Prospective study from a population-based cohort from Lausanne, Switzerland. Participants were categorized as non-users (no consumption at baseline and first follow-up), persistent users (consumption at baseline and follow-up), and occasional users (consumption either at baseline or follow-up). Incidence of CVD and of total mortality was assessed after the first follow-up.

**Results:**

Data from 4261 participants (57.4 ± 10.4 years, 55% females) was used. Median follow-up was 9 years (interquartile range 7.0–9.2) After multivariable analysis, no association was found between VMDS use and total mortality: hazard ratio and (95% confidence interval) 0.95 (0.71–1.28) and 0.83 (0.55–1.26) for occasional and persistent consumers, respectively, CVD mortality: 1.00 (0.47–2.11) and 1.30 (0.53–3.18), or CVD events: 0.96 (0.72–1.27) and 0.95 (0.64–1.42). Similar findings were obtained after inverse probability weighting, using only vitamin-mineral supplement users, or considering only participants at baseline. When CVD events were split into coronary heart disease (CHD) or stroke, persistent use of VMDS was associated with a higher risk of CHD in females: 3.12 (1.52–6.41), p = 0.002, but not in males, 0.25 (0.03–1.82), p = 0.171, p for interaction < 0.05. No association was found between VMDS use and incidence of stroke in both sexes.

**Conclusion:**

We found no association between vitamin and dietary supplement use and total or CVD mortality, or CVD events. The higher risk of CHD for persistent use in females should be further explored.

**Supplementary Information:**

The online version contains supplementary material available at 10.1007/s00394-025-03593-1.

## Introduction

Vitamin and dietary supplements are taken by a large fraction of the population, based on the belief of their beneficial effects on health. According to the 2011–2014 National Health and Nutrition Examination Survey, half (52%) of US adults reported using at least one dietary supplement and almost one-third (31%) reported using a vitamin-mineral supplement [1]. Vitamin and dietary supplements are also very common in the Swiss population, one-fifth of adults consuming them [2].

Despite the current belief that vitamin or dietary supplements improve health status, the evidence is different. Most studies on vitamin D failed to find any protective effect against cardiovascular disease (CVD) [3, 4], while a possible effect against cardiac failure was suggested [5]. No effect of vitamin C on CVD was found in a Cochrane review [6], but studies conducted in Asian populations showed a beneficial effect of vitamin C intake on the risk of stroke among smokers [7] and on total mortality [8]. Similarly, no effect of vitamin K [9] or vitamin B_3_ (niacin) in CVD prevention could be demonstrated [10], but the number and quality of the studies were low. In 2022, the US Preventive Task Force updated its recommendations for vitamin and dietary supplements issued in 2014 [11] and concluded that vitamin and mineral supplementation was associated with little or no benefit of preventing CVD or death [12, 13].

In previous publications, we have shown that vitamin supplement users have a better health [14] and a better dietary intake [15] than non-users, suggesting that the beneficial association between vitamin supplement use and health might be biased by a healthier lifestyle. We have also shown that vitamin supplement consumption does not lead to better fitness [16], and that the quality of some vitamin supplements in Switzerland failed to comply with the legal recommendations [17].

We have previously shown that vitamin supplement consumption is an irregular pattern, a sizable fraction of consumers discontinuing consumption and vice versa [2]. Whether long-term vitamin-mineral ± dietary supplement use has any effect on mortality or CVD has seldom been studied.

Hence, we aimed to assess if long-term vitamin-mineral ± dietary supplement consumption influences cardiovascular disease and mortality. We expected to replicate the lack of association reported previously [12, 13].

## Methods

### Study setting

The CoLaus|PsyCoLaus study is a population-based study investigating the epidemiology and genetic determinants of psychiatric and cardiovascular disease in Lausanne, Switzerland [18]. Briefly, a representative sample was collected through a simple, non-stratified random sampling of 19,830 individuals (35% of the source population) aged between 35 and 75. The baseline study was conducted between June 2003 and May 2006; the first follow-up was performed between April 2009 and September 2012; the second follow-up was performed between May 2014 and April 2017 and the third follow-up was performed between April 2018 and May 2021. Median follow-up time was 5.4 (average 5.6, range 4.5–8.8) years for the first follow-up, 10.7 (average 10.9, range 8.8–13.6) years for the second follow-up, and 14.5 (average 14.6, range 13.2–17.3) for the third follow-up.

### Death and cardiovascular events

During the follow-up period, first incident CVD events and deaths were prospectively collected and independently adjudicated according to established recommendations and similar definitions detailed elsewhere [19]. Details of the adjudication procedure are provided in the supplementary information.

### Vitamin / dietary supplement consumption

Participants reported all medicines and vitamin, or dietary supplements prescribed or bought over the counter. Vitamin-mineral and dietary supplement use were assessed for the baseline and the first follow-up. Vitamin and mineral supplements were defined according to the Swiss compendium (compendium.ch/home/fr, assessed June 2017). If the supplements were not listed in the Swiss compendium, further searches on the internet were conducted. Due to wide differences in the composition of Swiss vitamin and mineral supplements [17] and to inaccurate reporting (i.e., reporting “multivitamins from producer X” that manufactures six different types of multivitamins), it was not possible to assess the amounts of vitamins and minerals consumed by participants. Dietary supplements were defined as any other supplement that could not be considered as a VMS, such as plant extracts not considered as phytotherapy by the Swiss compendium, cod liver oil, shark cartilage or amino acids, for example. Participants were categorized as non-users (no consumption at baseline and follow-up), persistent users (consumption at baseline and follow-up), and occasional users (consumption either at baseline or follow-up) [2].

### Other covariates

A list of covariates potentially associated with vitamin or dietary supplement use was selected based on previous findings [14, 20]. Smoking was self-reported and categorized as never, former (irrespective of the time since quitting smoking) and current. Education was categorized into high (university), middle (high school) and low (apprenticeship + mandatory). Marital status was defined as living alone (single, divorced, widowed) or living with a partner. Nationality was defined as Swiss or other. Alcohol consumption was defined as consumer/not consumer. At baseline, physical activity was assessed by a single question querying the number of 20-min periods of vigorous physical activity performed per week, and participants were considered as sedentary if they responded “never”. In the first follow-up, physical activity was assessed by a questionnaire validated in the population of Geneva [21], and sedentary status was defined as spending more than 90% of the daily energy in activities below moderate- and high-intensity (defined as requiring at least 4 times the basal metabolic rate) [22]. Sedentary behaviour was preferred to other types of physical activity as it was the sole measure that was not overestimated when compared to accelerometry [23]. At baseline, no information regarding dietary intake was collected. In the first follow-up, dietary intake was assessed using a validated food frequency questionnaire validated in the population of Geneva [24] and the Mediterranean diet score according to Trichopoulou et al. [25] was computed to assess dietary quality.

Body weight and height were measured with participants barefoot and in light indoor clothes. Body weight was measured in kilograms to the nearest 100 g using a Seca® scale (Hamburg, Germany). Height was measured to the nearest 5 mm using a Seca® (Hamburg, Germany) height gauge. Body mass index was calculated and categorized as normal (< 25 kg/m^2^), overweight ≥ 25 and < 30 kg/m^2^) and obese ≥ 30 kg/m^2^).

Blood pressure (BP) was measured using an Omron® HEM-907 automated oscillometric sphygmomanometer after at least a 10-min rest in a seated position, and the average of the last two measurements was used. Hypertension was defined by a SBP ≥ 140 mm Hg or a DBP ≥ 90 mm Hg or presence of antihypertensive drug treatment.

Total cholesterol was assessed by CHOD-PAP, with maximum inter and intra-batch CVs of 1.6% and 1.7%, respectively. HDL-cholesterol was assessed by CHOD-PAP + PEG + cyclodextrin, with maximum inter and intra-batch CVs of 3.6% and 0.9%, respectively. Hypolipidemic drugs were considered if they were listed in the official list of drugs of Switzerland. Glucose was assessed by glucose dehydrogenase, with maximum inter and intra-batch CVs of 2.1% and 1.0%, respectively. Diabetes mellitus (DM) was defined as fasting plasma glucose ≥ 7.0 mmol/L and/or presence of oral hypoglycaemic or insulin treatment.

### Inclusion and exclusion criteria

Participants were considered as eligible if they participated in the first follow-up. Participants were excluded if they 1) did not participate in subsequent follow-up after the first one; 2) presented with previous CVD; 3) had missing data regarding supplements, and 4) had missing data for any covariate. As sedentary behaviour was missing in a sizable fraction of the sample, it was not considered as an exclusion criterion.

### Ethical statement

The institutional Ethics Committee of the University of Lausanne, which afterwards became the Ethics Commission of Canton Vaud (www.cer-vd.ch) approved the baseline CoLaus study (reference 16/03). The approval was renewed for the first (reference 33/09), the second (reference 26/14) and the third (reference PB_2018-000408) follow-ups. The approval for the entire CoLaus|PsyCoLaus study was confirmed in 2021 (reference PB_2018-00038, 239/09). The full decisions of the CER-VD can be obtained from the authors upon request. The study was performed in agreement with the Helsinki declaration and its former amendments, and in accordance with the applicable Swiss legislation. All participants gave their signed informed consent before entering the study.

### Statistical analysis

Statistical analyses were conducted using Stata v.16.1 (Stata Corp, College Station, TX, USA). Descriptive results were expressed as number of participants (percentage) for categorical variables and as average ± standard deviation or median [interquartile range] for continuous variables. Between-group comparisons for baseline characteristics were conducted using chi-square for categorical variables and student’s t-test, analysis of variance (ANOVA) or Kruskal–Wallis test for continuous variables.

Overall and CVD-related mortality and incidence of all CVD events were visualized as Kaplan-Meyer curves. Bivariate and multivariable associations between vitamin-mineral ± dietary supplement use and the outcomes of interest were assessed using Cox model for overall mortality and Fine-Gray competing risk model for cardiovascular disease, using non-cardiac mortality as competing event (21). Multivariable analyses were adjusted for sex, age, nationality, education (high, middle, low), marital status (living with a partner, living alone), smoking (never, former, current), body mass index categories (normal, overweight, obese), alcohol consumption (yes, no), Mediterranean diet score (continuous), hypertension (yes, no), diabetes (yes, no), and hypolipidemic drug treatment (yes, no). All confounders were evaluated at the first follow-up. Visual examination of the plots of the Scaled Schoenfeld residuals showed no violation of the proportional hazards assumption.

Several sensitivity analyses were conducted. The first one used inverse probability weighting to consider participants excluded from the main analysis. Briefly, logistic regression was used to estimate the likelihood of being included for each participant, and the inverse of predicted probability was then used for the survival analyses. The second one included only participants with sedentary behaviour data and adjusted for sedentary behaviour, 1) unweighted and 2) with inverse probability weighting. The third used only vitamin-mineral supplement (non) users, 1) unweighted; 2) with inverse probability weighting, and 3) including only participants with sedentary behaviour data and adjusting for sedentary behaviour. The fourth used baseline data; in this case, only users and non-users were considered, and no adjustment on dietary quality was performed. The fifth stratified the analysis by sex, and an interaction term was included between vitamin-mineral ± dietary supplement use (never, alternate, and persistent) and sex. The sixth separated coronary heart disease and stroke, and analyses were conducted overall and stratified by sex, with an interaction term as before. Statistical significance was considered for a two-sided test with p < 0.05.

## Results

### Sample characteristics

Out of the 6′733 initial participants, 5′064 were considered as eligible, and 803 were excluded. The reasons for exclusion are summarized in supplementary Fig. 1 and the characteristics of included and excluded participants are listed in supplementary Table 1. Excluded participants were older, more frequently male, of lesser education, current smokers, obese, and presenting with hypertension or diabetes.

### *Association of vitamin-mineral* ± *dietary supplement use with overall mortality and cardiovascular disease*

Overall, two thirds (66.8%) of the participants did not consume vitamin-mineral ± dietary supplements between the baseline and the first follow-up, and less than one in eleven (8.7%) reported consuming vitamin-mineral ± dietary supplements at both study periods. Their clinical characteristics are summarized in Table 1. Never consumers were younger, less frequently female, born in Switzerland, living alone, of normal weight, took less frequently hypolipidemic drugs, had lower levels of total and HDL cholesterol andwere more frequently current smokers.

**Table 1:** Characteristics of participants according to vitamin-mineral ± dietary supplement use, CoLaus|PsyCoLaus study, Lausanne, Switzerland

	Never	Occasional	Persistent	P-value
N (%)	2846 (66.8)	1044 (24.5)	371 (8.7)	
Age (years)	56.1 ± 10.2	58.9 ± 10.4	63.9 ± 9.4	< 0.001
Female (%)	1313 (46.1)	733 (70.2)	301 (81.1)	< 0.001
Born in Switzerland (%)	1735 (61.0)	706 (67.6)	273 (73.6)	< 0.001
Educational level (%)				0.029
High	646 (22.7)	228 (21.9)	70 (18.9)	
Middle	726 (25.5)	287 (27.5)	123 (33.2)	
Low	1472 (51.8)	528 (50.6)	178 (48.0)	
Living alone (%)	1120 (39.4)	508 (48.7)	194 (52.3)	< 0.001
Smoking status (%)				< 0.001
Never	1158 (40.7)	466 (44.6)	150 (40.4)	
Former	1034 (36.3)	388 (37.2)	164 (44.2)	
Current	654 (23.0)	190 (18.2)	57 (15.4)	
Body mass index (kg/m^2^)	26.4 ± 4.5	25.5 ± 4.5	24.8 ± 4.5	< 0.001
BMI categories (%)				< 0.001
Normal	1168 (41.0)	542 (51.9)	212 (57.1)	
Overweight	1174 (41.3)	351 (33.6)	118 (31.8)	
Obese	504 (17.7)	151 (14.5)	41 (11.1)	
Alcohol consumption (%)	2375 (83.7)	861 (82.6)	292 (78.9)	0.062
Sedentary behaviour (%) §	1371 (56.6)	493 (54.6)	209 (64.1)	0.012
Mediterranean score	3.9 ± 1.5	4.0 ± 1.5	4.0 ± 1.4	0.416
Hypertension (%)	1125 (39.5)	375 (35.9)	151 (40.7)	0.089
Systolic BP (mm Hg)	126 ± 17	124 ± 18	127 ± 18	0.032
Diastolic BP (mm Hg)	79 ± 11	77 ± 10	76 ± 11	< 0.001
Diabetes (%)	300 (10.5)	77 (7.4)	35 (9.4)	0.012
Hypolipidemic drugs (%)	397 (14.0)	172 (16.5)	83 (22.4)	< 0.001
Total cholesterol (mmol/L)	5.72 ± 1.00	5.76 ± 1.02	5.86 ± 1.05	0.040
HDL cholesterol (mmol/L)	1.60 ± 0.45	1.74 ± 0.49	1.82 ± 0.47	< 0.001

BMI, body mass index, BP, blood pressure

§ sample sizes are 2421, 903 and 326 for never, occasional, and persistent, respectively. Results are expressed as number of participants (percentage) for categorical variables and as average ± standard deviation for continuous variables. Between-group comparisons performed using chi-square for categorical variables and analysis of variance for continuous variables

**Fig. 1:**
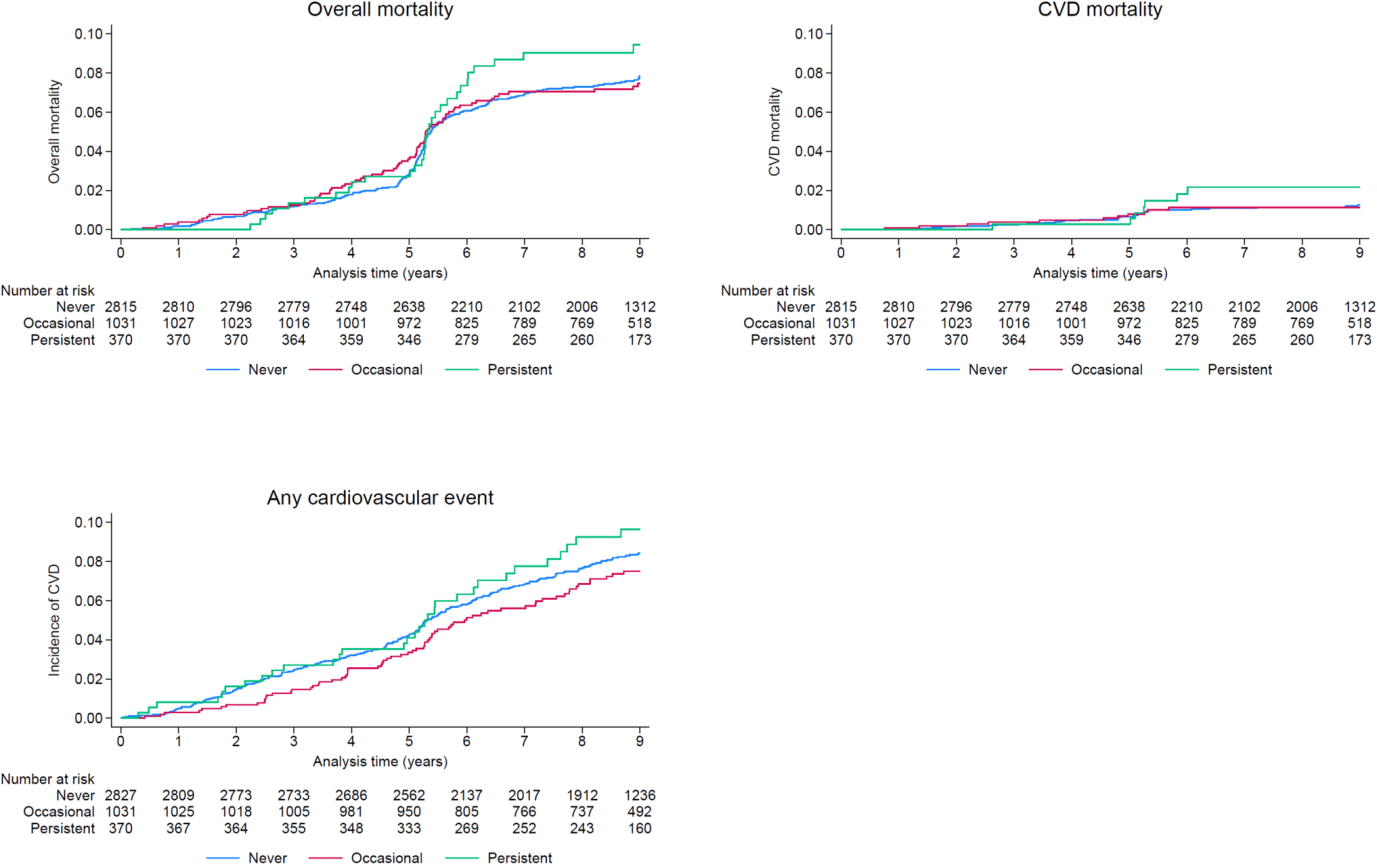
overall and CVD-related mortality and all cardiovascular events according to never, alternate, and persistent vitamin mineral ± dietary supplement use, CoLaus|PsyCoLaus study, Lausanne, Switzerland

After a median 9 years of follow-up (interquartile range: 7.0–9.2), 326 deaths (of which 52 due to CVD) and 321 CVD events were recorded. Total mortality and incidence of cardiovascular disease according to vitamin-mineral ± dietary supplement use are indicated in Fig. 1 and Table 2 and the associations between vitamin-mineral ± dietary supplement use and overall mortality and CVD are summarized in Table 3. Compared to never users, no statistical differences were found for occasional or persistent users for all outcomes considered.

**Table 2:** Total mortality and incidence of cardiovascular disease according to vitamin-mineral ± dietary supplement use, CoLaus|PsyCoLaus study, Lausanne, Switzerland

	Person-years	Failures	Rate (95% CI)
Mortality			
Never	22,778.0	215	9.4 (8.3–10.8)
Occasional	8424.5	78	9.3 (7.4–11.6)
Persistent	2951.6	33	11.2 (7.9–15.7)
Cardiovascular death			
Never	22,778.0	33	1.4 (1.0–2.0)
Occasional	8424.5	12	1.4 (0.8–2.5)
Persistent	2951.6	7	2.4 (1.1–5.0)
Cardiovascular disease			
Never	22,244.6	216	9.7 (8.5–11.1)
Occasional	8267.5	73	8.8 (7.0–11.1)
Persistent	2862.6	32	11.2 (7.9–15.8)

Results are expressed as mortality or incidence rate per 1000 person-years

**Table 3:** association between vitamin-mineral ± dietary supplement use and total mortality or incidence of cardiovascular disease, CoLaus|PsyCoLaus study, Lausanne, Switzerland

	Bivariate	P-value	Multivariable	P-value
Mortality				
Never	1 (ref)		1 (ref)	
Occasional	0.98 (0.75—1.27)	0.860	0.95 (0.71—1.28)	0.744
Persistent	1.23 (0.85—1.77)	0.276	0.83 (0.55—1.26)	0.392
CVD mortality				
Never	1 (ref)		1 (ref)	
Occasional	1.00 (0.51—1.93)	0.989	1.00 (0.47—2.11)	0.993
Persistent	1.68 (0.74—3.79)	0.215	1.30 (0.53—3.18)	0.570
CVD events				
Never	1 (ref)		1 (ref)	
Occasional	0.91 (0.70—1.19)	0.498	0.96 (0.72—1.27)	0.764
Persistent	1.17 (0.81—1.69)	0.414	0.95 (0.64—1.42)	0.797

CVD, cardiovascular disease. Results are expressed as bivariate or multivariable-adjusted hazard ratios and (95% confidence intervals). Analysis conducted using Cox regression for total mortality and CVD events, and Fine-Gray competing risk regression for CVD mortality. Multivariable analysis adjusted for sex, age, nationality, education (high, middle, low), marital status (living with a partner, living alone), smoking (never, former, current), body mass index categories (normal, overweight, obese), alcohol consumption (yes, no), Mediterranean diet score (continuous), hypertension (yes, no), diabetes (yes, no) and hypolipidemic drug treatment (yes, no)

### Sensitivity analyses

The results of the analysis using inverse probability weighting are provided in supplementary Table 2. A trend towards a higher mortality risk for persistent users was observed on bivariate analysis, but no association was found after multivariable analysis. Restricting the analysis to participants with sedentary behaviour data led to similar findings (supplementary Table 3).

The clinical characteristics according to vitamin-mineral supplement use are summarized in supplementary Table 4. Overall, most participants did not consume vitamin-mineral supplements between the two survey periods. Never consumers shared the same characteristics as in Table 1. Total mortality and incidence of cardiovascular disease according to vitamin-mineral supplement use are provided in supplementary Table 5, and the associations between vitamin-mineral supplement use and overall mortality and CVD are summarized in supplementary Table 6. Compared to never users, no statistical differences were found for occasional or persistent users for all outcomes considered. Similar findings were obtained when inverse probability weighting was applied (supplementary Table 7) or when the analysis was restricted to participants with sedentary behaviour data (supplementary Table 8).

Of the 6733 participants with baseline data, 1375 (20.4%) were excluded for the analysis between vitamin-mineral ± dietary supplement use and outcomes starting at baseline. The characteristics of included and excluded participants are summarized in supplementary Table 9 and the characteristics of participants according to vitamin-mineral ± dietary supplement use are indicated in supplementary Table 10. After a median follow-up of 14.4 years (interquartile range 11.2–14.7 years), 687 deaths (of which 116 due to CVD) and 575 CVD events occurred (supplementary Table 11). The associations between vitamin-mineral ± dietary supplement use and overall mortality and CVD are summarized in supplementary Table 12. No significant association was found between vitamin-mineral ± dietary supplement use and all outcomes.

When analyses were stratified by sex, a lower risk of overall mortality was found for persistent users among females, while no association was found for males, p for interaction 0.011. No association between vitamin-mineral + dietary supplement use and CVD mortality or CVD events was found (supplementary tables 13 to 16).

When events were split into coronary heart disease or stroke, no association was found for the overall sample (supplementary tables 17 and 18). When analysis was stratified by sex, persistent use was associated with a higher risk of coronary heart disease in females (supplementary Table 19, p for interaction < 0.05) while no association was found for males. No association between vitamin-mineral + dietary supplement use and stroke was found for both sexes (supplementary Table 19).

## Discussion

In this population-based, prospective study, we found no association between the use of multivitamin and dietary supplements and total or CVD mortality or CVD, in people that have no vitamin deficit. The lower overall mortality risk of persistent use among females should be further explored.

### *Association of vitamin-mineral* ± *dietary supplement use with overall mortality and cardiovascular disease*

Most studies so far assessed the effect of vitamin-mineral ± dietary supplements on CVD or mortality using a single time point assessment of supplement consumption. Our results add further information to the prior literature regarding the effect of supplements on CVD. Indeed, several studies recently published concluded that vitamin and mineral supplementation was associated with little or no benefit in preventing cancer, CVD, and death [12, 26]. The O’Connor’s Vitamin and Mineral Supplements for the Primary Prevention of Cardiovascular Disease and Cancer study, found in 2022 that there was little to no association in vitamin and mineral supplementation and preventing CVD and death [12]. Similar patterns were seen in the Sesso’s Multivitamins in the prevention of cancer and cardiovascular disease study, proving that multivitamin-multimineral use neither significantly affected all-cause mortality nor reduced the incidence of total cancer [26]. The United States Preventive Services Task Force recommendations concluded that “the current evidence is insufficient to assess the balance of benefits and harms of the use of multivitamin supplements for the prevention of CVD or cancer” [13].

The supposedly beneficial effects of vitamin-mineral ± dietary supplements on CVD or on mortality are likely explained by the healthier lifestyle of users [14], such as a healthier diet [15]. For instance, the Leisure World Cohort Study found no association between antioxidant vitamin use and total mortality, the initially observed associations being considerably attenuated after adjustment for confounders [27]. Although a Chinese study reported a protective effect of antioxidant vitamin use on CVD but not on cancer mortality [28], a meta-analysis of seventy-eight randomized trials found no evidence to support antioxidant supplements for primary or secondary prevention, with beta-carotene and vitamin E even showing a tendency to increase mortality [29]. Overall, our results confirm mostly of the existing literature that neither regular nor intermittent vitamin-mineral ± dietary supplements are associated with decreased risk of total mortality, CVD mortality, or CVD events. The lower overall mortality risk observed in females could be due to a lower incidence of cancer, as it has been suggested by the SU.VI.MAX study [30]. It could also be due to a better dietary intake and a better health management among females relative to males. Conversely, when the analysis was split into coronary heart disease and stroke, a higher risk of coronary heart disease was found among persistent users in females, but not in males, while no association was found with stroke. The reasons for such an association can only be hypothesized. Females may use vitamin or dietary supplements to manage their cardiovascular risk factors [31], thus leading to a lesser control. Another hypothesis would be that some dietary supplements either possess cardiovascular modifying properties or are contaminated by potentially cardiohazardous products [32]. Dietary supplements can also interact with prescribed drugs [33], and excess consumption of vitamin-mineral supplements might lead to body accumulation and possible intoxication, with potentially deleterious effects [34, 35]. Overall, our results suggest that, in females, persistent use of vitamin-mineral ± dietary supplements might bring some benefits regarding mortality, at the expense of an increased risk of coronary heart disease. It would be important that other studies could replicate our findings.

### Implications for clinical practice

These findings confirm the lack of association between multivitamin and dietary supplement use and CVD events or overall mortality in males. Neither the US [12, 13] nor the European [36] guidelines recommend multivitamin and/or dietary supplements as a preventive measure against CVD. Hence, for the time being, doctors should refrain from prescribing multivitamin and/or dietary supplements as a preventive measure against CVD.

### Strengths and limitations

This is one of the few prospective studies conducted in a European country, which assessed the effect of vitamin-mineral supplement use, with and without association with dietary supplements. To our knowledge, it is also the only one that assessed the effect of persistent, occasional, and never use of vitamin-mineral ± dietary supplement use on CVD events, CVD- and total mortality.

Our study also has some limitations. First, it was conducted in a single location, and results might not be generalizable to other settings due to the differences in vitamin-mineral or dietary supplements. Still, it is unlikely that the differences in vitamin-mineral supplements be of such magnitude that they will change the findings. Second, it was not possible to precisely assess the composition of all vitamin-mineral and dietary supplements, due to lack of precise information provided by the participants, and to the large variability of vitamin-mineral supplements available in Switzerland [17]. Still, our results suggest that, irrespective of their composition, vitamin-mineral and dietary supplements do not protect from CVD, a finding also reported elsewhere [12, 13]. Third, and due to the previous limitation, it was not possible to assess if persistent users always consumed the same supplement or had switched to another one. Fourth, it was not possible to confirm if persistent users consumed the supplements throughout the whole follow-up period. Still, our results indicate that a higher vitamin-mineral ± dietary supplement use does not protect from CVD. Finally, a sizable fraction of the initial sample had to be excluded; even though the results were similar after inverse probability weighting, results should be considered with caution. Still, they replicate findings from previous studies [12, 13].

## Conclusion

In this population-based prospective study, we found no association between vitamin-mineral ± dietary supplement use and total or CVD mortality or CVD. Consumption of vitamin-mineral ± dietary supplements to prevent CVD is not recommended.

## Supplementary Information

Below is the link to the electronic supplementary material.

Supplementary file1 (PPTX 41 KB)

Supplementary file2 (DOCX 104 KB)

## Data Availability

The data of CoLaus|PsyCoLaus study used in this article cannot be fully shared as they contain potentially sensitive personal information on participants. According to the Ethics Committee for Research of the Canton of Vaud, sharing these data would be a violation of the Swiss legislation with respect to privacy protection. However, coded individual-level data that do not allow researchers to identify participants are available upon request to researchers who meet the criteria for data sharing of the CoLaus|PsyCoLaus Datacenter (CHUV, Lausanne, Switzerland). Any researcher affiliated to a public or private research institution who complies with the CoLaus|PsyCoLaus standards can submit a research application to research.colaus@chuv.ch or research.psycolaus@chuv.ch. Proposals requiring baseline data only, will be evaluated by the baseline (local) Scientific Committee (SC) of the CoLaus and PsyCoLaus studies. Proposals requiring follow-up data will be evaluated by the follow-up (multicentric) SC of the CoLaus|PsyCoLaus cohort study. Detailed instructions for gaining access to the CoLaus|PsyCoLaus data used in this study are available at www.colaus-psycolaus.ch/professionals/how-to-collaborate/.

## Declarations

### Declaration of generative artificial intelligence (AI) in scientific writing

The authors indicate that they did not use generative AI when writing their manuscript.
