# Supplementary material for: Vitamin and dietary supplements are not associated with total or cardiovascular mortality in Switzerland: the CoLaus|PsyCoLaus prospective study
Source: Eur J Nutr. 2025 Feb 1;64(2):81. doi: 10.1007/s00394-025-03593-1 (PMC11787243; doi:10.1007/s00394-025-03593-1)
Supplement: Supplementary file 1 — Supplementary file1 (PPTX 41 KB) [file 394_2025_3593_MOESM1_ESM.pptx]

## Slide 1
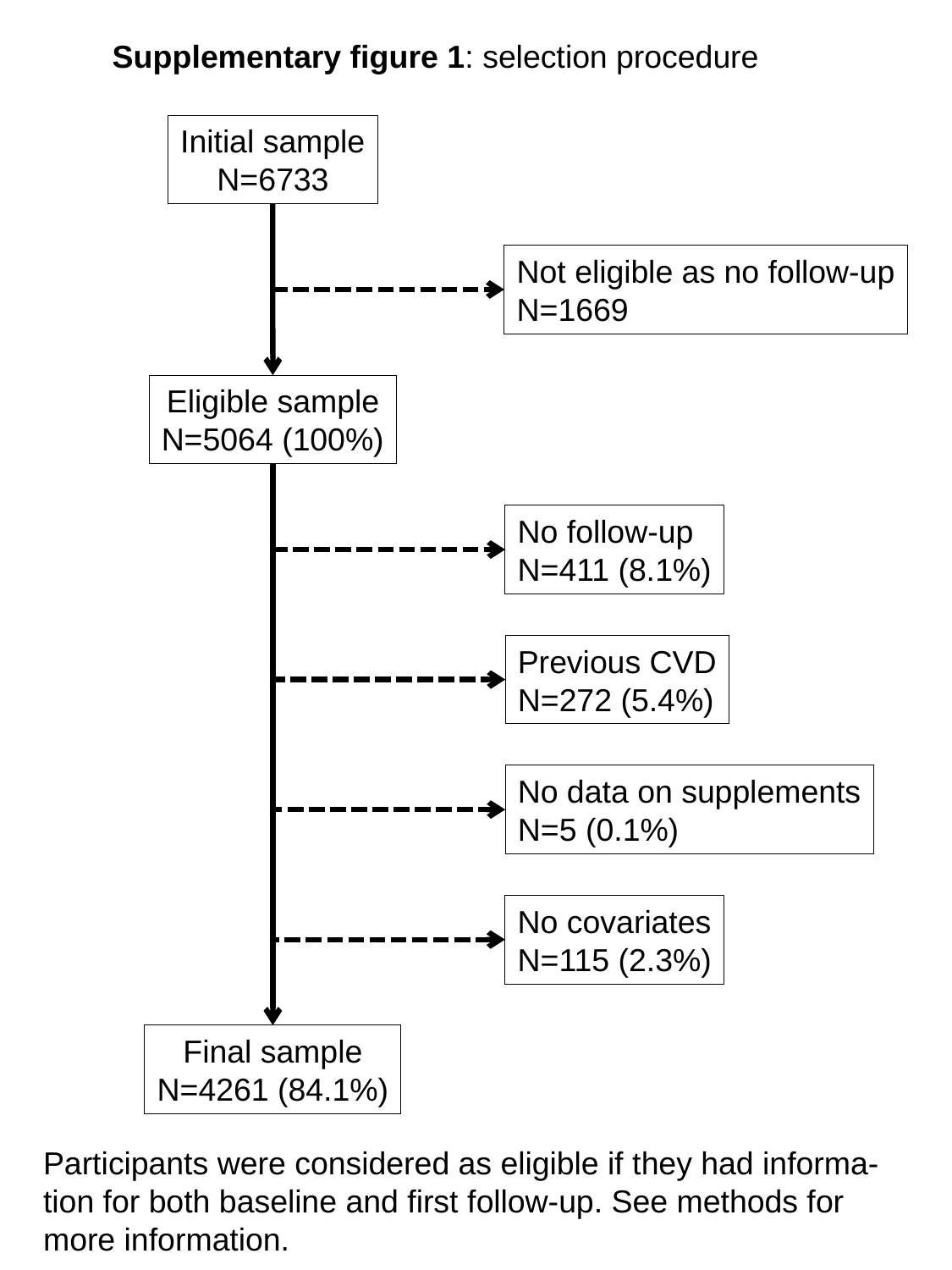

Supplementary figure 1: selection procedure
Initial sample
N=6733
Not eligible as no follow-up
N=1669
Eligible sample
N=5064 (100%)
No follow-up
N=411 (8.1%)
Previous CVD
N=272 (5.4%)
No data on supplements
N=5 (0.1%)
No covariates
N=115 (2.3%)
Final sample
N=4261 (84.1%)
Participants were considered as eligible if they had informa-tion for both baseline and first follow-up. See methods for more information.
