# Supplementary material for: Vitamin and dietary supplements are not associated with total or cardiovascular mortality in Switzerland: the CoLaus|PsyCoLaus prospective study
Source: Eur J Nutr. 2025 Feb 1;64(2):81. doi: 10.1007/s00394-025-03593-1 (PMC11787243; doi:10.1007/s00394-025-03593-1)
Supplement: Supplementary file 2 — Supplementary file2 (DOCX 104 KB) [file 394_2025_3593_MOESM2_ESM.docx]

**Supplementary information**

## Clinical data collection in the CoLaus Study

Prevalent and incident CV events were recorded through a stepwise process.

- First, relevant medical records of participants who declared, during the baseline and/or follow-up examinations, to have presented a CVD and/or CVD-related procedure during their lifetime, including MI, angina pectoris, stroke, arrhythmia, cardiomyopathy, coronarography and/or percutaneous transluminal coronary angioplasty (PTCA) and/or coronary stenting, coronary artery bypass grafting (CABG) and peripheral artery disease (PAD), were collected. The records were collected from general practitioners, cardiologists, neurologists and/or hospitals (as appropriate), and encompassed medical and/or surgical notes, laboratory, radiological, echocardiographic and electrocardiographic reports. If necessary, the original coronarography (angiogram) and brain CT/MRI exams were collected.
- Second, to retrieve events that may not have been mentioned during interviews, the central medical database of the University Hospital of Lausanne, which is the main community hospital in the catchment area of the study, was searched. Participants with hospital records were identified by cross-checking with administrative data and events of interest were detected using the following ICD-10 (*International Classification of diseases, Tenth Edition*) codes: I20.0, I21.-, I22.-, I24.-, I25.1-, I25.2-, I25.5, I25.6, I25.8, I25.9, I61.-, I62.-, I63.-, I64, I69.1, I69.2, I69.3, I69.4, I69.8, and G45.-.
- Third, death was established using the population register of the city where the participant was living in case of returned mail, absence of response when calling and/or indication from a relative. Information on cause of death was sequentially collected from: 1) general practitioners; 2) medical database of the hospital where the death occurred (either in Switzerland or abroad); 3) database of the pre-hospital emergency care unit of the City of Lausanne; 4) database of the University Centres of Forensic Medicine of Lausanne and Geneva; 5) official death certificates from the Swiss federal office of statistics; 6) verbal autopsy with a relative of the dead participant, if all previous steps failed.

## Adjudication of CV events

### **Coronary events**

All coronary-related events were adjudicated by two cardiologists based upon an international expert consensus document.[1] For this study, we defined major coronary events as a composite outcome including: i) acute coronary syndromes (ACS) (acute myocardial infarction (AMI) or unstable angina) and ii) symptomatic stable angina followed by a revascularization procedure, either by percutaneous coronary intervention (PCI) or by coronary artery bypass grafting (CABG). Iatrogenic events caused by a medical procedure (angioplasty, stenting, or coronary artery bypass surgery) were not considered. The following criteria were applied:

1. Definite AMI, including ST-segment elevation myocardial infarction (STEMI) and Non-ST-segment elevation myocardial infarction and (NSTEMI) was defined in the presence of at least one of the following criteria:
2. Detection of rise/fall of Troponin I or T with at least one value above the 99^th^ percentile of the upper reference limit together with evidence of myocardial ischaemia with *at least one* of the following:
   - Symptoms of ischaemia
   - ECG changes indicative of new ischaemia (new ST-T changes or new left bundle branch block)
   - Development of pathological Q waves in the ECG
   - Echocardiographic evidence of new segmental abnormality
3. Sudden, unexpected cardiac death, involving cardiac arrest, often with symptoms suggestive of myocardial ischaemia, and accompanied by presumably new ST elevation, or new left bundle branch block

**and/or**

- - Evidence of fresh thrombus by coronary angiography and/or at autopsy

**but**

- - Occurring before blood samples could be obtained, or at a time before the appearance of cardiac biomarkers in the blood (Troponin I or T).

1. Due to missing information on cardiac biomarkers, probable AMI was defined using the following criteria:
2. Symptoms of ischaemia

**and**

1. ECG changes: new ST-T changes, new left bundle branch block or new pathological Q waves

**and/or**

1. Coronary angiography followed by percutaneous or surgical revascularization

**and/or**

1. Echocardiographic evidence of new segmental abnormality
2. Unstable angina was defined using the following criteria (used by Cardiobase/SPUM)**:**
3. Symptoms of ischaemia
4. Hospitalization or ambulatory care
5. Negative troponin values
6. Change in medication after hospital discharge or during ambulatory care (betablocker, calcium blocker, nitro-derivatives, molsidomine, nicorandil)
7. Coronary artery disease (CAD) events corresponded to participants who presented with typical symptoms (stable angina) and underwent either percutaneous (PTCA ± stenting) or surgical (CABG) revascularizations, unless these procedures were directly related to an AMI. When the exact date of the event could not be traced, the date of the revascularization was used (n=4). The following criteria were used to define CAD:
8. Symptoms of ischaemia

**and**

1. Coronary angiography followed by percutaneous or surgical revascularization

**without:**

- - Acute context
  - ECG changes compatible with AMI
  - Modification in medication (-> unstable angina).

1. Detection of rise/fall of troponin I or T

### **Strokes and transient ischemic attacks**

Fatal and nonfatal strokes were adjudicated by one neurologist in participants who presented non-traumatic and rapidly progressing focal or global disturbances of cerebral function lasting ≥24h.[2, 3]

Ischaemic origin was based upon normal imaging or imaging (CT and/or MRI) showing a recent lesion of ischaemic nature and compatible with the clinical presentation. Ischaemic strokes with haemorrhagic conversion were also listed here. Haemorrhagic origin was based upon imaging (CT and/or MRI) showing the presence of intracerebral, intraventricular and/or subarachnoid blood of presumed spontaneous occurrence and compatible with the clinical presentation. Transient ischaemic attacks were defined upon rapidly developing focal or global disturbances of cerebral function of presumed vascular origin and lasting <24 hours.[4]

Stroke was defined according to WHO definition [5], i.e. as rapidly developing clinical signs of focal or global disturbance of cerebral function of presumed vascular origin lasting ≥ 24 hours

- *Ischemic* : normal imaging or imaging showing recent lesion of ischemic nature correponding to clinical syndrome. Ischemic strokes with conversion to haemorrhage are listed here.
- *Haemorrhagic* : intracerebral and/or intraventricular and/or subarachnoid blood of presumed spontaneous appearance and corresponding to clinical syndrome.
- Transient ischemic attack [4] was defined as rapidly developing clinical signs of focal or global disturbance of presumed vascular origin lasting < 24 hours.
- *With normal imaging*
- *With imaging showing recent lesion* of presumed ischemic nature and correponding to clinical syndrome.

**Remarks:**

- Not due to traumatism and not due to transformation of an ischemic stroke. Non-traumatic symptomatic subarachnoid haemorrhagic is considered a haemorrhagic stroke.
- Traditionally, the TIA definition was purely clinical (complete disappearance of symptoms within 24 hours). In a new proposal [4], a TIA with pathological imaging is considered a « stroke », and a TIA without pathological imaging is considered a « TIA » (= a transient episode of neurological dysfunction caused by focal brain, spinal cord, or retinal ischemia, without acute infarction). The problem with this new definition is that it depends strongly on the type of imaging used.
- An ophthalmologist’s exam related to the event is mandatory to exclude other diseases and to confirm the arterial occlusion.

The following cerebrovascular diseases were not considered as outcome events:

1. Spinal cord ischemia
2. Retinal artery occlusion (central retinal artery or branch retinal artery) : rapidly developed clinical signs of disturbance of retinal function of presumed vascular origin lasting ≥ 24 hours.[2]
3. *Amaurosis fugax* of presumed arterial origin: rapidly developed clinical signs of disturbance of retinal function of presumed vascular origin lasting ≥ 24 hours.[2]
4. Cerebral sinus vein thrombosis.
5. Intracranial vascular malformations with other than ischemic or haemorrhagic manifestations.

### **Cardiovascular deaths**

Deaths were adjudicated by two internists and were classified as cardiac, vascular and non-cardiovascular; deaths from cerebrovascular origin were already defined as previously mentioned. Cardiac deaths, including fatal MI and cardiac sudden deaths, were based upon the same criteria as non-fatal MI.[1] Vascular deaths encompassed aortic dissection, valvular heart disease, fatal arrhythmia and cardiac failure. Non-cardiovascular deaths included all other diagnoses not listed above (e.g. accident, infection/sepsis, cancer, pulmonary embolism, suicide, etc.). Undetermined deaths were defined as deaths that occurred outside hospital with or without witness. Unless another diagnosis could be established, they were listed as cardiac if their origin could be reasonably attributable to a coronary event, for example by the presence of typical symptoms just before death and/or personal history (presence of ≥2 traditional CV risk factors and/or ACS or CAD).

**Supplementary table 1** – Characteristics at follow-up 1 of excluded and included participants, CoLaus|PsyCoLaus study, Lausanne, Switzerland.

|  | Included | Excluded | P-value |
| --- | --- | --- | --- |
| N | 4261 | 803 |  |
| Age (years) | 57.4 ± 10.4 | 59.5 ± 11.0 | <0.001 |
| Female (%) | 2347 (55.1) | 360 (44.8) | <0.001 |
| Born in Switzerland (%) | 2714 (63.7) | 470 (58.5) | 0.005 |
| Educational level (%) |  |  | <0.001 |
| High | 944 (22.2) | 135 (16.9) |  |
| Middle | 1136 (26.7) | 170 (21.2) |  |
| Low | 2178 (51.2) | 496 (61.9) |  |
| Living alone (%) | 1822 (42.8) | 380 (47.3) | 0.017 |
| Smoking status (%) |  |  | 0.002 |
| Never | 1774 (41.6) | 261 (35.0) |  |
| Former | 1586 (37.2) | 297 (39.8) |  |
| Current | 901 (21.2) | 188 (25.2) |  |
| Body mass index (kg/m^2^) | 26.0 ± 4.5 | 27.1 ± 4.9 | <0.001 |
| BMI categories (%) |  |  | 0.002 |
| Normal | 1922 (45.1) | 258 (35.0) |  |
| Overweight | 1643 (38.6) | 317 (43.0) |  |
| Obese | 696 (16.3) | 163 (22.0) |  |
| Alcohol consumption (%) | 3528 (83.0) | 595 (79.2) | 0.012 |
| Sedentary behaviour (%) § | 2073 (56.8) | 332 (62.5) | 0.013 |
| Mediterranean score | 4.0 ± 1.5 | 3.9 ± 1.5 | 0.868 |
| Hypertension (%) | 1651 (38.8) | 445 (56.1) | <0.001 |
| Diabetes (%) | 412 (9.7) | 127 (16.2) | <0.001 |

BMI, body mass index. § sample size is 3650 for included participants. Results are expressed as number of participants (percentage) for categorical variables and as average±standard deviation for continuous variables. Between-group comparisons performed using chi-square for categorical variables and student’s t-test for continuous variables.

**Supplementary table 2 –** association between vitamin-mineral ± dietary supplement use and total mortality or incidence of cardiovascular disease, CoLaus|PsyCoLaus study, Lausanne, Switzerland, using inverse probability weighting.

|  | Bivariate | P-value | Multivariate | P-value |
| --- | --- | --- | --- | --- |
| Mortality |  |  |  |  |
| Never | 1 (ref) |  | 1 (ref) |  |
| Alternate | 0.99 (0.74 - 1.32) | 0.925 | 0.86 (0.62 - 1.18) | 0.343 |
| Persistent | 1.50 (1.00 - 2.25) | 0.051 | 0.91 (0.59 - 1.42) | 0.682 |
| CVD mortality |  |  |  |  |
| Never | 1 (ref) |  | 1 (ref) |  |
| Alternate | 0.94 (0.46 - 1.90) | 0.861 | 0.78 (0.34 - 1.78) | 0.548 |
| Persistent | 1.98 (0.81 - 4.86) | 0.135 | 1.19 (0.43 - 3.31) | 0.734 |
| CVD events |  |  |  |  |
| Never | 1 (ref) |  | 1 (ref) |  |
| Alternate | 0.99 (0.74 - 1.33) | 0.958 | 0.95 (0.70 - 1.29) | 0.755 |
| Persistent | 1.45 (0.97 - 2.17) | 0.072 | 1.02 (0.68 - 1.55) | 0.914 |

CVD, cardiovascular disease. Results are expressed as bivariate or multivariate-adjusted hazard ratios and (95% confidence intervals). Analysis conducted using Cox regression for total mortality and CVD events, and Fine-Gray competing risk regression for CVD mortality. Multivariate analysis adjusted for sex, age, nationality, education (high, middle, low), marital status (living with a partner, living alone), smoking (never, former, current), body mass index categories (normal, overweight, obese), alcohol consumption (yes, no), Mediterranean diet score (continuous), hypertension (yes, no), diabetes (yes, no), and hypolipidemic drug treatment (yes, no). Inverse probability weighting considering exclusion criteria.

**Supplementary table 3 –** association between vitamin-mineral ± dietary supplement use and total mortality or incidence of cardiovascular disease, CoLaus|PsyCoLaus study, Lausanne, Switzerland, only participants with sedentary behaviour data.

|  | Bivariate | P-value | Multivariate | P-value |
| --- | --- | --- | --- | --- |
| Mortality |  |  |  |  |
| Never | 1 (ref) |  | 1 (ref) |  |
| Alternate | 1.04 (0.78 - 1.40) | 0.775 | 0.97 (0.71 - 1.32) | 0.830 |
| Persistent | 1.23 (0.82 - 1.87) | 0.318 | 0.86 (0.55 - 1.33) | 0.497 |
| CVD mortality |  |  |  |  |
| Never | 1 (ref) |  | 1 (ref) |  |
| Alternate | 1.07 (0.49 - 2.31) | 0.870 | 0.85 (0.38 - 1.94) | 0.707 |
| Persistent | 2.37 (1.02 - 5.53) | 0.046 | 1.36 (0.54 - 3.45) | 0.513 |
| CVD events |  |  |  |  |
| Never | 1 (ref) |  | 1 (ref) |  |
| Alternate | 1.02 (0.77 - 1.35) | 0.899 | 1.04 (0.77 - 1.39) | 0.813 |
| Persistent | 1.34 (0.92 - 1.97) | 0.131 | 1.04 (0.69 - 1.57) | 0.837 |

CVD, cardiovascular disease. Results are expressed as bivariate or multivariate-adjusted hazard ratios and (95% confidence intervals). Analysis conducted using Cox regression for total mortality and CVD events, and Fine-Gray competing risk regression for CVD mortality. Multivariate analysis adjusted for sex, age, nationality, education (high, middle, low), marital status (living with a partner, living alone), smoking (never, former, current), body mass index categories (normal, overweight, obese), alcohol consumption (yes, no), Mediterranean diet score (continuous), sedentary behaviour (yes, no), hypertension (yes, no), diabetes (yes, no), and hypolipidemic drug treatment (yes, no). Inverse probability weighting considering exclusion criteria.

**Supplementary table 4** – Characteristics of participants according to vitamin-mineral supplement use, CoLaus|PsyCoLaus study, Lausanne, Switzerland.

|  | Never | Alternate | Persistent | P-value |
| --- | --- | --- | --- | --- |
| N | **3002** | **930** | **329** |  |
| Age (years) | 56.2 ± 10.2 | 58.9 ± 10.4 | 64.2 ± 9.2 | <0.001 |
| Female (%) | 1432 (47.7) | 652 (70.1) | 263 (79.9) | <0.001 |
| Born in Switzerland (%) | 1843 (61.4) | 627 (67.4) | 244 (74.2) | <0.001 |
| Educational level (%) |  |  |  | 0.058 |
| High | 672 (22.4) | 210 (22.6) | 62 (18.8) |  |
| Middle | 773 (25.8) | 254 (27.3) | 109 (33.1) |  |
| Low | 1555 (51.8) | 465 (50.1) | 158 (48.1) |  |
| Living alone (%) | 1208 (40.2) | 447 (48.1) | 167 (50.8) | <0.001 |
| Smoking status (%) |  |  |  | 0.001 |
| Never | 1232 (41.1) | 412 (44.3) | 130 (39.5) |  |
| Former | 1091 (36.3) | 349 (37.5) | 146 (44.4) |  |
| Current | 679 (22.6) | 169 (18.2) | 53 (16.1) |  |
| Body mass index (kg/m^2^) | 26.4 ± 4.5 | 25.3 ± 4.4 | 24.9 ± 4.6 | <0.001 |
| BMI categories (%) |  |  |  | <0.001 |
| Normal | 1251 (41.7) | 485 (52.2) | 186 (56.5) |  |
| Overweight | 1214 (40.4) | 324 (34.8) | 105 (31.9) |  |
| Obese | 537 (17.9) | 121 (13.0) | 38 (11.6) |  |
| Alcohol consumption (%) | 2508 (83.8) | 762 (82.0) | 258 (78.7) | 0.042 |
| Sedentary behaviour (%) § | 1445 (56.6) | 441 (54.8) | 187 (64.0) | 0.022 |
| Mediterranean score | 3.9 ± 1.5 | 4.0 ± 1.5 | 4.0 ± 1.4 | 0.764 |
| Hypertension (%) | 1186 (39.5) | 330 (35.5) | 135 (41.0) | 0.060 |
| Systolic BP (mm Hg) | 126 ± 17 | 125 ± 18 | 126 ± 18 | 0.229 |
| Diastolic BP (mm Hg) | 79 ± 11 | 77 ± 10 | 75 ± 11 | <0.001 |
| Diabetes (%) | 310 (10.3) | 70 (7.5) | 32 (9.7) | 0.041 |
| Hypolipidemic drugs (%) | 421 (14.0) | 151 (16.2) | 80 (24.3) | <0.001 |
| Total cholesterol (mmol/L) | 5.72 ± 1.01 | 5.78 ± 1.01 | 5.83 ± 1.05 | 0.066 |
| HDL cholesterol (mmol/L) | 1.61 ± 0.45 | 1.75 ± 0.49 | 1.79 ± 0.46 | <0.001 |

BMI, body mass index; BP, blood pressure. § sample sizes are 2553, 805 and 292 for never, alternate, and persistent, respectively. Results are expressed as number of participants (percentage) for categorical variables and as average±standard deviation for continuous variables. Between-group comparisons performed using chi-square for categorical variables and analysis of variance for continuous variables.

**Supplementary table 5** – Total mortality and incidence of cardiovascular disease according to vitamin-mineral supplement use, CoLaus|PsyCoLaus study, Lausanne, Switzerland.

|  | Person-years | Failures | Rate (95% CI) |
| --- | --- | --- | --- |
| Mortality |  |  |  |
| Never | 24,080.7 | 224 | 9.3 (8.2 - 10.6) |
| Alternate | 7472.1 | 72 | 9.6 (7.6 - 12.1) |
| Persistent | 2601.4 | 30 | 11.5 (8.1 - 16.5) |
| CVD mortality |  |  |  |
| Never | 24,080.7 | 33 | 1.4 (1.0 - 1.9) |
| Alternate | 7472.1 | 13 | 1.7 (1.0 - 3.0) |
| Persistent | 2601.4 | 6 | 2.3 (1.0 - 5.1) |
| CVD events |  |  |  |
| Never | 23,520.3 | 226 | 9.6 (8.4 - 10.9) |
| Alternate | 7330.9 | 66 | 9 (7.1 - 11.5) |
| Persistent | 2523.4 | 29 | 11.5 (8.0 - 16.5) |

CI, confidence interval. Results are expressed as mortality or incidence rate per 1000 person-years.

**Supplementary table 6 –** association between vitamin-mineral supplement use and total mortality or incidence of cardiovascular disease, CoLaus|PsyCoLaus study, Lausanne, Switzerland.

|  | Bivariate | P-value | Multivariate | P-value |
| --- | --- | --- | --- | --- |
| Mortality |  |  |  |  |
| Never | 1 (ref) |  | 1 (ref) |  |
| Alternate | 1.04 (0.79 - 1.35) | 0.796 | 1.05 (0.78 - 1.41) | 0.766 |
| Persistent | 1.29 (0.88 - 1.88) | 0.197 | 0.86 (0.56 - 1.33) | 0.503 |
| CVD mortality |  |  |  |  |
| Never | 1 (ref) |  | 1 (ref) |  |
| Alternate | 1.29 (0.68 - 2.46) | 0.436 | 1.30 (0.64 - 2.65) | 0.464 |
| Persistent | 1.72 (0.72 - 4.09) | 0.224 | 1.34 (0.53 - 3.43) | 0.538 |
| CVD events |  |  |  |  |
| Never | 1 (ref) |  | 1 (ref) |  |
| Alternate | 0.94 (0.72 - 1.24) | 0.673 | 0.98 (0.73 - 1.31) | 0.888 |
| Persistent | 1.21 (0.82 - 1.79) | 0.326 | 0.96 (0.63 - 1.45) | 0.846 |

CVD, cardiovascular disease. Results are expressed as bivariate or multivariate-adjusted hazard ratios and (95% confidence intervals). Analysis conducted using Cox regression for total mortality and CVD events, and Fine-Gray competing risk regression for CVD mortality. Multivariate analysis adjusted for sex, age, nationality, education (high, middle, low), marital status (living with a partner, living alone), smoking (never, former, current), body mass index categories (normal, overweight, obese), alcohol consumption (yes, no), Mediterranean diet score (continuous), hypertension (yes, no), diabetes (yes, no) and hypolipidemic drug treatment (yes, no).

**Supplementary table 7 –** association between vitamin supplement use and total mortality or incidence of cardiovascular disease, CoLaus|PsyCoLaus study, Lausanne, Switzerland, using inverse probability weighting.

|  | Bivariate | P-value | Multivariate | P-value |
| --- | --- | --- | --- | --- |
| Mortality |  |  |  |  |
| Never | 1 (ref) |  | 1 (ref) |  |
| Alternate | 1.06 (0.79 - 1.42) | 0.708 | 0.98 (0.71 - 1.35) | 0.906 |
| Persistent | 1.50 (0.98 - 2.29) | 0.060 | 0.93 (0.58 - 1.48) | 0.758 |
| CVD mortality |  |  |  |  |
| Never | 1 (ref) |  | 1 (ref) |  |
| Alternate | 1.29 (0.65 - 2.59) | 0.468 | 1.08 (0.50 - 2.34) | 0.849 |
| Persistent | 1.93 (0.73 - 5.05) | 0.183 | 1.21 (0.41 - 3.61) | 0.733 |
| CVD events |  |  |  |  |
| Never | 1 (ref) |  | 1 (ref) |  |
| Alternate | 1.03 (0.76 - 1.39) | 0.856 | 0.97 (0.71 - 1.34) | 0.875 |
| Persistent | 1.42 (0.94 - 2.17) | 0.099 | 0.98 (0.63 - 1.51) | 0.913 |

CVD, cardiovascular disease. Results are expressed as bivariate or multivariate-adjusted hazard ratios and (95% confidence intervals). Analysis conducted using Cox regression for total mortality and CVD events, and Fine-Gray competing risk regression for CVD mortality. Multivariate analysis adjusted for sex, age, nationality, education (high, middle, low), marital status (living with a partner, living alone), smoking (never, former, current), body mass index categories (normal, overweight, obese), alcohol consumption (yes, no), Mediterranean diet score (continuous), hypertension (yes, no), diabetes (yes, no) and hypolipidemic drug treatment (yes, no). Inverse probability weighting considering exclusion criteria.

**Supplementary table 8 –** association between vitamin-mineral supplement use and total mortality or incidence of cardiovascular disease, CoLaus|PsyCoLaus study, Lausanne, Switzerland, only participants with sedentary behaviour data.

|  | Bivariate | P-value | Multivariate | P-value |
| --- | --- | --- | --- | --- |
| Mortality |  |  |  |  |
| Never | 1 (ref) |  | 1 (ref) |  |
| Alternate | 1.12 (0.84 - 1.51) | 0.438 | 1.10 (0.80 - 1.51) | 0.546 |
| Persistent | 1.24 (0.80 - 1.92) | 0.326 | 0.89 (0.57 - 1.41) | 0.632 |
| CVD mortality |  |  |  |  |
| Never | 1 (ref) |  | 1 (ref) |  |
| Alternate | 1.43 (0.68 - 3.01) | 0.351 | 1.13 (0.53 - 2.42) | 0.748 |
| Persistent | 2.41 (0.98 - 5.92) | 0.056 | 1.43 (0.55 - 3.75) | 0.465 |
| CVD events |  |  |  |  |
| Never | 1 (ref) |  | 1 (ref) |  |
| Alternate | 1.03 (0.78 - 1.38) | 0.817 | 1.04 (0.77 - 1.41) | 0.778 |
| Persistent | 1.36 (0.92 - 2.03) | 0.128 | 1.04 (0.68 - 1.60) | 0.840 |

CVD, cardiovascular disease. Results are expressed as bivariate or multivariate-adjusted hazard ratios and (95% confidence intervals). Analysis conducted using Cox regression for total mortality and CVD events, and Fine-Gray competing risk regression for CVD mortality. Multivariate analysis adjusted for sex, age, nationality, education (high, middle, low), marital status (living with a partner, living alone), smoking (never, former, current), body mass index categories (normal, overweight, obese), alcohol consumption (yes, no), Mediterranean diet score (continuous), sedentary behaviour (yes, no), hypertension (yes, no), diabetes (yes, no) and hypolipidemic drug treatment (yes, no).

**Supplementary table 9** – Characteristics at baseline of excluded and included participants at baseline, CoLaus|PsyCoLaus study, Lausanne, Switzerland.

|  | Included | Excluded | P-value |
| --- | --- | --- | --- |
| N | 5358 | 1375 |  |
| Age (years) | 52.8 ± 10.7 | 52.1 ± 10.7 | 0.061 |
| Female (%) | 2899 (54.1) | 645 (46.9) | <0.001 |
| Born in Switzerland (%) | 3387 (63.2) | 644 (46.9) | <0.001 |
| Educational level (%) |  |  | <0.001 |
| High | 1096 (20.5) | 224 (16.4) |  |
| Middle | 1353 (25.3) | 272 (19.9) |  |
| Low | 2906 (54.3) | 868 (63.6) |  |
| Living alone (%) | 1770 (33.0) | 444 (32.5) | 0.684 |
| Smoking status (%) |  |  | 0.070 |
| Never | 2181 (40.7) | 551 (40.3) |  |
| Former | 1765 (32.9) | 418 (30.5) |  |
| Current | 1412 (26.4) | 400 (29.2) |  |
| Body mass index (kg/m^2^) | 25.6 ± 4.5 | 26.5 ± 4.7 | <0.001 |
| BMI categories (%) |  |  | <0.001 |
| Normal | 2685 (50.1) | 552 (40.2) |  |
| Overweight | 1919 (35.8) | 543 (39.6) |  |
| Obese | 754 (14.1) | 277 (20.2) |  |
| Alcohol consumption (%) | 4223 (78.8) | 998 (72.9) | <0.001 |
| Sedentary behaviour (%) | 1816 (33.9) | 561 (40.8) | <0.001 |
| Hypertension (%) | 1881 (35.1) | 619 (45.3) | <0.001 |
| Diabetes (%) | 315 (5.9) | 121 (8.9) | <0.001 |

BMI, body mass index. Results are expressed as number of participants (percentage) for categorical variables and as average±standard deviation for continuous variables. Between-group comparisons performed using chi-square for categorical variables and student’s t-test for continuous variables.

**Supplementary table 10** –Characteristics of participants according to vitamin-mineral ± dietary supplement use at baseline, CoLaus|PsyCoLaus study, Lausanne, Switzerland.

|  | Nonusers | Users | P-value |
| --- | --- | --- | --- |
| N | 4235 | 1123 |  |
| Age (years) | 51.9 ± 10.6 | 55.9 ± 10.7 | <0.001 |
| Female (%) | 2089 (49.3) | 810 (72.1) | <0.001 |
| Born in Switzerland (%) | 2590 (61.2) | 797 (71.0) | <0.001 |
| Educational level (%) |  |  | 0.104 |
| High | 875 (20.7) | 221 (19.7) |  |
| Middle | 1042 (24.6) | 311 (27.7) |  |
| Low | 2316 (54.7) | 590 (52.6) |  |
| Living alone (%) | 1315 (31.1) | 455 (40.5) | <0.001 |
| Smoking status (%) |  |  | 0.001 |
| Never | 1720 (40.6) | 461 (41.1) |  |
| Former | 1356 (32.0) | 409 (36.4) |  |
| Current | 1159 (27.4) | 253 (22.5) |  |
| Body mass index (kg/m^2^) | 25.8 ± 4.5 | 24.9 ± 4.4 | <0.001 |
| BMI categories (%) |  |  | <0.001 |
| Normal | 2046 (48.3) | 639 (56.9) |  |
| Overweight | 1557 (36.8) | 362 (32.2) |  |
| Obese | 632 (14.9) | 122 (10.9) |  |
| Alcohol consumption (%) | 3342 (78.9) | 881 (78.5) | 0.725 |
| Sedentary behaviour (%) | 1517 (35.8) | 299 (26.6) | <0.001 |
| Hypertension (%) | 1493 (35.3) | 388 (34.6) | 0.661 |
| Systolic BP (mm Hg) | 128 ± 18 | 127 ± 18 | 0.462 |
| Diastolic BP (mm Hg) | 79 ± 11 | 78 ± 11 | <0.001 |
| Diabetes (%) | 259 (6.1) | 56 (5.0) | 0.153 |
| Hypolipidemic drugs (%) | 389 (9.2) | 181 (16.1) | <0.001 |
| Total cholesterol (mmol/L) | 5.58 ± 1.02 | 5.62 ± 1.03 | 0.237 |
| HDL cholesterol (mmol/L) | 1.62 ± 0.44 | 1.74 ± 0.45 | <0.001 |

BMI, body mass index; BP, blood pressure. Results are expressed as number of participants (percentage) for categorical variables and as average±standard deviation for continuous variables. Between-group comparisons performed using chi-square for categorical variables and student’s t-test for continuous variables.

**Supplementary table 11** – Total mortality and incidence of cardiovascular disease according to vitamin-mineral ± dietary supplement use at baseline, CoLaus|PsyCoLaus study, Lausanne, Switzerland.

|  | Person-years | Failures | Rate (95% CI) |
| --- | --- | --- | --- |
| Mortality |  |  |  |
| Nonusers | 54’743.1 | 539 | 9.8 (9 - 10.7) |
| Users | 14’598.3 | 148 | 10.1 (8.6 - 11.9) |
| CVD mortality |  |  |  |
| Nonusers | 54’743.1 | 87 | 1.6 (1.3 - 2.0) |
| Users | 14’598.3 | 29 | 2.0 (1.4 - 2.9) |
| CVD events |  |  |  |
| Nonusers | 52’993.4 | 458 | 8.6 (7.9 - 9.5) |
| Users | 14’139.5 | 117 | 8.3 (6.9 - 9.9) |

CI, confidence interval. Results are expressed as mortality or incidence rate per 1000 person-years.

**Supplementary table 12 –** association between vitamin-mineral ± dietary supplement use at baseline and total mortality or incidence of cardiovascular disease, CoLaus|PsyCoLaus study, Lausanne, Switzerland.

|  | Bivariate | P-value | Multivariate | P-value |
| --- | --- | --- | --- | --- |
| Mortality |  |  |  |  |
| Nonusers | 1 (ref) |  | 1 (ref) |  |
| Users | 1.05 (0.88 - 1.27) | 0.566 | 0.93 (0.77 - 1.13) | 0.486 |
| CVD mortality |  |  |  |  |
| Nonusers | 1 (ref) |  | 1 (ref) |  |
| Users | 1.26 (0.83 - 1.91) | 0.283 | 1.14 (0.74 - 1.77) | 0.553 |
| CVD events |  |  |  |  |
| Nonusers | 1 (ref) |  | 1 (ref) |  |
| Users | 0.96 (0.78 - 1.18) | 0.698 | 0.94 (0.76 - 1.17) | 0.592 |

CVD, cardiovascular disease. Results are expressed as bivariate or multivariate-adjusted hazard ratios and (95% confidence intervals). Analysis conducted using Cox regression for total mortality and CVD events, and Fine-Gray competing risk regression for CVD mortality. Multivariate analysis adjusted for sex, age, nationality, education (high, middle, low), marital status (living with a partner, living alone), smoking (never, former, current), body mass index categories (normal, overweight, obese), alcohol consumption (yes, no), sedentary behaviour (yes, no), hypertension (yes, no), diabetes (yes, no) and hypolipidemic drug treatment (yes, no).

**Supplementary table 13** – Total mortality and incidence of cardiovascular disease according to vitamin-mineral ± dietary supplement use, CoLaus|PsyCoLaus study, Lausanne, Switzerland. Results stratified by sex.

|  | Women | | | Men | | |
| --- | --- | --- | --- | --- | --- | --- |
|  | **Person-years** | **Failures** | **Rate (95% CI)** | **Person-years** | **Failures** | **Rate (95% CI)** |
| Mortality |  |  |  |  |  |  |
| Never | 10,541.7 | 74 | 7 (5.6 - 8.8) | 12,236.3 | 141 | 11.5 (9.8 - 13.6) |
| Occasional | 5938.0 | 44 | 7.4 (5.5 - 10) | 2486.5 | 34 | 13.7 (9.8 - 19.1) |
| Persistent | 2419.9 | 19 | 7.9 (5 - 12.3) | 531.7 | 14 | 26.3 (15.6 - 44.5) |
| Cardiovascular death |  |  |  |  |  |  |
| Never | 10,541.7 | 9 | 0.9 (0.4 - 1.6) | 12,236.3 | 24 | 2.0 (1.3 - 2.9) |
| Occasional | 5938.0 | 8 | 1.3 (0.7 - 2.7) | 2486.5 | 4 | 1.6 (0.6 - 4.3) |
| Persistent | 2419.9 | 4 | 1.7 (0.6 - 4.4) | 531.7 | 3 | 5.6 (1.8 - 17.5) |
| Cardiovascular disease |  |  |  |  |  |  |
| Never | 10,411.6 | 61 | 5.9 (4.6 - 7.5) | 11,833.0 | 155 | 13.1 (11.2 - 15.3) |
| Occasional | 5847.5 | 41 | 7.0 (5.2 - 9.5) | 2420.0 | 32 | 13.2 (9.4 - 18.7) |
| Persistent | 2347.9 | 28 | 11.9 (8.2 - 17.3) | 514.6 | 4 | 7.8 (2.9 - 20.7) |

Results are expressed as mortality or incidence rate per 1000 person-years.

**Supplementary table 14** **–** association between vitamin-mineral ± dietary supplement use and total mortality or incidence of cardiovascular disease, CoLaus|PsyCoLaus study, Lausanne, Switzerland. Results stratified by sex.

|  | Females | | | | Males | | | |
| --- | --- | --- | --- | --- | --- | --- | --- | --- |
|  | **Bivariate** | **P-value** | **Multivariable** | **P-value** | **Bivariate** | **P-value** | **Multivariable** | **P-value** |
| Mortality |  |  |  |  |  |  |  |  |
| Never | 1 (ref) |  | 1 (ref) |  | 1 (ref) |  | 1 (ref) |  |
| Occasional | 1.04 (0.72 - 1.52) | 0.821 | 0.81 (0.55 - 1.19) | 0.278 | 1.20 (0.82 - 1.74) | 0.348 | 1.16 (0.79 - 1.70) | 0.450 |
| Persistent | 1.14 (0.69 - 1.90) | 0.601 | 0.52 (0.31 - 0.88) | 0.014 | 2.44 (1.41 - 4.23) | 0.001 | 1.45 (0.82 - 2.56) | 0.201 |
| CVD mortality |  |  |  |  |  |  |  |  |
| Never | 1 (ref) |  | 1 (ref) |  | 1 (ref) |  | 1 (ref) |  |
| Occasional | 1.57 (0.6 - 4.08) | 0.356 | 1.17 (0.39 - 3.51) | 0.782 | 0.81 (0.29 - 2.32) | 0.701 | 0.75 (0.26 - 2.18) | 0.596 |
| Persistent | 1.92 (0.59 - 6.25) | 0.277 | 1.01 (0.28 - 3.69) | 0.984 | 2.92 (0.89 - 9.60) | 0.078 | 1.47 (0.52 - 4.13) | 0.469 |
| CVD events |  |  |  |  |  |  |  |  |
| Never | 1 (ref) |  | 1 (ref) |  | 1 (ref) |  | 1 (ref) |  |
| Occasional | 1.19 (0.8 - 1.78) | 0.378 | 0.95 (0.64 - 1.43) | 0.812 | 1.00 (0.69 - 1.47) | 0.981 | 0.99 (0.67 - 1.45) | 0.945 |
| Persistent | 2.05 (1.31 - 3.21) | 0.002 | 1.17 (0.73 - 1.87) | 0.513 | 0.60 (0.22 - 1.63) | 0.320 | 0.44 (0.16 - 1.19) | 0.106 |

CVD, cardiovascular disease. Results are expressed as bivariate or multivariable-adjusted hazard ratios and (95% confidence intervals). Analysis conducted using Cox regression for total mortality and CVD events, and Fine-Gray competing risk regression for CVD mortality. Multivariable analysis adjusted for age, nationality, education (high, middle, low), marital status (living with a partner, living alone), smoking (never, former, current), body mass index categories (normal, overweight, obese), hypertension (yes, no), diabetes (yes, no) and hypolipidemic drug treatment (yes, no).

**Supplementary table 15** – Total mortality and incidence of cardiovascular disease according to vitamin-mineral ± dietary supplement use at baseline, CoLaus|PsyCoLaus study, Lausanne, Switzerland. Results stratified by sex.

|  | Females | | | Males | | |
| --- | --- | --- | --- | --- | --- | --- |
|  | **Person-years** | **Failures** | **Rate (95% CI)** | **Person-years** | **Failures** | **Rate (95% CI)** |
| Mortality |  |  |  |  |  |  |
| Nonusers | 27,147.4 | 203 | 7.5 (6.5 - 8.6) | 27,595.7 | 336 | 12.2 (10.9 - 13.5) |
| Users | 10,641.9 | 81 | 7.6 (6.1 - 9.5) | 3956.4 | 67 | 16.9 (13.3 - 21.5) |
| CVD mortality |  |  |  |  |  |  |
| Nonusers | 27,147.4 | 25 | 0.9 (0.6 - 1.4) | 27,595.7 | 62 | 2.2 (1.8 - 2.9) |
| Users | 10,641.9 | 17 | 1.6 (1.0 - 2.6) | 3956.4 | 12 | 3.0 (1.7 - 5.3) |
| CVD events |  |  |  |  |  |  |
| Nonusers | 26,699.0 | 142 | 5.3 (4.5 - 6.3) | 26,294.3 | 316 | 12 (10.8 - 13.4) |
| Users | 10,336.2 | 74 | 7.2 (5.7 - 9.0) | 3803.3 | 43 | 11.3 (8.4 - 15.2) |

CI, confidence interval. Results are expressed as mortality or incidence rate per 1000 person-years.

**Supplementary table 17** – Total mortality and incidence of coronary heart disease or stroke according to vitamin-mineral ± dietary supplement use, CoLaus|PsyCoLaus study, Lausanne, Switzerland. Results presented for the entire sample or stratified by sex.

|  | Coronary heart disease | | | Stroke | | |
| --- | --- | --- | --- | --- | --- | --- |
|  | **Person-years** | **Failures** | **Rate (95% CI)** | **Person-years** | **Failures** | **Rate (95% CI)** |
| All participants |  |  |  |  |  |  |
| Never | 22,448.8 | 108 | 4.8 (4.0 - 5.8) | 22,562.3 | 97 | 4.3 (3.5 - 5.2) |
| Occasional | 8,323.9 | 39 | 4.7 (3.4 - 6.4) | 8,367.9 | 30 | 3.6 (2.5 - 5.1) |
| Persistent | 2,894.4 | 19 | 6.6 (4.2 - 10.3) | 2,919.8 | 11 | 3.8 (2.1 - 6.8) |
| Females |  |  |  |  |  |  |
| Never | 10,496.9 | 21 | 2.0 (1.3 - 3.1) | 10,462.0 | 34 | 3.2 (2.3 - 4.5) |
| Occasional | 5,878.5 | 21 | 3.6 (2.3 - 5.5) | 5,906.8 | 18 | 3.0 (1.9 - 4.8) |
| Persistent | 2,368.4 | 18 | 7.6 (4.8 - 12.1) | 2,399.5 | 9 | 3.8 (2.0 - 7.2) |
| Males |  |  |  |  |  |  |
| Never | 11,951.9 | 87 | 7.3 (5.9 - 9.0) | 12,100.3 | 63 | 5.2 (4.1 - 6.7) |
| Occasional | 2,445.4 | 18 | 7.4 (4.6 - 11.7) | 2,461.2 | 12 | 4.9 (2.8 - 8.6) |
| Persistent | 526.0 | 1 | 1.9 (0.3 - 13.5) | 520.4 | 2 | 3.8 (1.0 - 15.4) |

Results are expressed as mortality or incidence rate per 1000 person-years.

**Supplementary table 18** **–** association between vitamin-mineral ± dietary supplement use and coronary heart disease or stroke, CoLaus|PsyCoLaus study, Lausanne, Switzerland.

|  | Coronary heart disease | P-value | Stroke | P-value |
| --- | --- | --- | --- | --- |
| Unadjusted |  |  |  |  |
| Never | 1 (ref) |  | 1 (ref) |  |
| Occasional | 0.98 (0.68 - 1.41) | 0.900 | 0.83 (0.55 - 1.25) | 0.381 |
| Persistent | 1.39 (0.85 - 2.26) | 0.186 | 0.88 (0.47 - 1.64) | 0.678 |
| Model 1 |  |  |  |  |
| Never | 1 (ref) |  | 1 (ref) |  |
| Occasional | 1.09 (0.74 - 1.62) | 0.656 | 0.81 (0.53 - 1.24) | 0.335 |
| Persistent | 1.32 (0.77 - 2.26) | 0.315 | 0.63 (0.33 - 1.21) | 0.162 |
| Model 2 |  |  |  |  |
| Never | 1 (ref) |  | 1 (ref) |  |
| Occasional | 1.04 (0.68 - 1.58) | 0.871 | 0.87 (0.55 - 1.37) | 0.540 |
| Persistent | 1.48 (0.86 - 2.53) | 0.153 | 0.68 (0.34 - 1.33) | 0.257 |
| Model 3 |  |  |  |  |
| Never | 1 (ref) |  | 1 (ref) |  |
| Occasional | 1.26 (0.84 - 1.89) | 0.256 | 0.86 (0.56 - 1.34) | 0.511 |
| Persistent | 1.60 (0.93 - 2.77) | 0.092 | 0.61 (0.31 - 1.21) | 0.156 |

Results are expressed as bivariate or multivariable-adjusted hazard ratios and (95% confidence intervals). Analysis conducted using Cox regression. Model 1 adjusted for sex, age, nationality, education (high, middle, low), marital status (living with a partner, living alone), smoking (never, former, current), body mass index categories (normal, overweight, obese), hypertension (yes, no), diabetes (yes, no) and hypolipidemic drug treatment (yes, no). Model 2: same as model 1, with inverse probability weighting. Model 3: same as model 1, plus adjusting on sedentary behaviour.

**Supplementary table 19** **–** association between vitamin-mineral ± dietary supplement use and coronary heart disease or stroke, CoLaus|PsyCoLaus study, Lausanne, Switzerland. Results stratified by sex.

|  | Females | | | | Males | | | |
| --- | --- | --- | --- | --- | --- | --- | --- | --- |
|  | **CHD** | **P-value** | **Stroke** | **P-value** | **CHD** | **P-value** | **Stroke** | **P-value** |
| Unadjusted |  |  |  |  |  |  |  |  |
| Never | 1 (ref) |  | 1 (ref) |  | 1 (ref) |  | 1 (ref) |  |
| Occasional | 1.78 (0.97 - 3.26) | 0.062 | 0.94 (0.53 - 1.66) | 0.830 | 1.00 (0.60 - 1.67) | 0.993 | 0.94 (0.51 - 1.74) | 0.837 |
| Persistent | 3.85 (2.05 - 7.22) | <0.001 | 1.16 (0.56 - 2.41) | 0.696 | 0.26 (0.04 - 1.9) | 0.187 | 0.74 (0.18 - 3.03) | 0.676 |
| Model 1 |  |  |  |  |  |  |  |  |
| Never | 1 (ref) |  | 1 (ref) |  | 1 (ref) |  | 1 (ref) |  |
| Occasional | 1.53 (0.80 - 2.92) | 0.202 | 0.75 (0.42 - 1.34) | 0.330 | 0.93 (0.54 - 1.58) | 0.775 | 0.94 (0.50 - 1.77) | 0.851 |
| Persistent | 2.55 (1.27 - 5.10) | 0.008 | 0.64 (0.30 - 1.38) | 0.256 | 0.19 (0.03 - 1.41) | 0.106 | 0.51 (0.12 - 2.14) | 0.359 |
| Model 2 |  |  |  |  |  |  |  |  |
| Never | 1 (ref) |  | 1 (ref) |  | 1 (ref) |  | 1 (ref) |  |
| Occasional | 1.52 (0.78 - 2.97) | 0.219 | 0.81 (0.44 - 1.51) | 0.507 | 0.76 (0.42 - 1.38) | 0.362 | 1.04 (0.54 - 1.99) | 0.911 |
| Persistent | 2.69 (1.38 - 5.25) | 0.004 | 0.69 (0.32 - 1.51) | 0.355 | 0.09 (0.01 - 0.70) | 0.022 | 0.49 (0.11 - 2.13) | 0.344 |
| Model 3 |  |  |  |  |  |  |  |  |
| Never | 1 (ref) |  | 1 (ref) |  | 1 (ref) |  | 1 (ref) |  |
| Occasional | 1.92 (0.97 - 3.77) | 0.060 | 0.81 (0.45 - 1.47) | 0.492 | 1.02 (0.59 - 1.75) | 0.949 | 0.94 (0.48 - 1.82) | 0.845 |
| Persistent | 3.12 (1.52 - 6.41) | 0.002 | 0.56 (0.25 - 1.25) | 0.156 | 0.25 (0.03 - 1.82) | 0.171 | 0.64 (0.15 - 2.70) | 0.545 |

CHD, coronary heart disease. Results are expressed as bivariate or multivariable-adjusted hazard ratios and (95% confidence intervals). Analysis conducted using Cox regression. Model 1 adjusted for age, nationality, education (high, middle, low), marital status (living with a partner, living alone), smoking (never, former, current), body mass index categories (normal, overweight, obese), hypertension (yes, no), diabetes (yes, no) and hypolipidemic drug treatment (yes, no). Model 2: same as model 1, with inverse probability weighting. Model 3: same as model 1, plus adjusting on sedentary behaviour. Test for interaction between persistent vitamin-mineral ± dietary supplement use and sex: p=0.014 for Model 1, p=0.002 for Model 2, and p=0.017 for Model 3.
